# Identification of Plasma Protein Biomarkers and Drug Targets for Hematologic Malignancies by Proteome-wide Mendelian Randomization

**DOI:** 10.7150/jca.115044

**Published:** 2025-09-03

**Authors:** Tao Pan, Jiyue Zhang, Xiaomin Wang, Yuqin Song

**Affiliations:** 1 Key Laboratory of Carcinogenesis and Translational Research (Ministry of Education/Beijing), Department of Lymphoma, Peking University Cancer Hospital & Institute, Beijing, 100142, China.; 2 Key Laboratory of Carcinogenesis and Translational Research (Ministry of Education/Beijing), Laboratory of Lymphoma Translational Research, Peking University Cancer Hospital & Institute, Beijing, 100142, China.

**Keywords:** hematologic malignancies, plasma protein, Mendelian randomization, drug target

## Abstract

**Background:** It has been reported that the proteome in blood was an important source for biomarker and therapeutic target discovery. However, up to now, few proteomes have been identified with the risk of hematologic malignancies.

**Methods:** Genome-wide association studies (GWASs) including 3,083 plasma proteins are based on data from 54,219 people in the UK Biobank Pharma Proteomics Project (UKB-PPP) and 35,559 individuals from Iceland (deCODE). Genetic correlations with 33 hematologic malignancies were derived from the FinnGen cohort and the UK Biobank Data. Further studies, including Bayesian colocalization, protein-protein interaction assessment, pathway enrichment analysis, and drug target evaluation, were performed to enhance knowledge and identify prospective therapeutic targets for 33 hematologic cancers.

**Results**: Our study indicated that 86 potential plasma proteins may have a substantial causal association with the incidence of 33 hematological tumors, such as BCL2, NFKB1, PARP1, and TNFRSF14. There are 18 proteins with strong evidence of genetic co-localization and 9 proteins with moderate support from colocalization analysis. Out of the 86 proteins, 51 have druggable targets, and 26 were identified as targets for current or prospective pharmaceuticals.

**Conclusion**: Our research revealed numerous significant proteins linked to the likelihood of hematologic malignancies. It may elucidate protein-mediated processes of hematological tumors and provide prospective treatment options for individuals with these conditions.

## Introduction

Hematologic malignancies, including conditions such as leukemia, lymphoma, and myeloma, arise from the alteration of hematopoietic cells 1. They are characterized by the unregulated expansion of hematopoietic cells, resulting in significant disturbances in hematopoietic and immunological processes, greatly impacting the patient's quality of life and overall survival rate 2. The primary treatment modalities for hematological tumors consist of chemotherapy, radiation, and stem cell transplantation 3. Despite advancements in patient prognosis via these treatments, they often present limits, including restricted effectiveness, considerable adverse effects, and the development of resistance. Consequently, investigating the molecular causes of hematological malignancies is essential for improving patient prognosis. Recent advancements in comprehending the molecular foundation of hematological tumors have generated interest in discovering biomarkers that may function as therapeutic targets, with several plasma proteins already recognized as prospective biomarkers and therapeutic argots 4. The identification of atypical protein expression patterns in the plasma of individuals with malignant hematological disorders has initiated new research opportunities. These proteins provide insights into illness development and prognosis and may also serve as effective targets for innovative therapeutic approaches, possibly enhancing clinical tomes 5. Through the use of genetic variation as an instrumental variable, Mendelian randomization (MR) provides a robust framework for conducting an investigation into the causal relationship that exists between plasma proteins and the consequences of sickness 6. This approach allows researchers to alleviate confounding biases and reverse causality problems often identified in observational studies 7.

The primary aim of this project is to comprehensively discover and assess plasma protein biomarkers linked to hematological malignancies via a Mendelian randomization framework. GWAS of levels of circulating proteins could identify protein quantitative trait loci (pQTLs). Combining pQTLs and disease-variant associations can examine the causative effects of the proteins on the disease through Mendelian randomisation. This work is important due to recent improvements in proteomics technology that facilitate the detection of low-abundance proteins in plasma. These improvements may provide new insights into the diagnostic and prognostic capabilities of plasma proteins, hence facilitating the development of precision medicine techniques for managing hematological tumors. This work included the collection of data from two extensive proteome-wide GWAS and 33 blood malignancy GWAS, followed by a proteome-wide MR analysis to comprehensively identify circulating protein biomarkers linked to the risk of blood malignancies. Given that MR alone may be inadequate for identifying genuine proteins associated with cancer causative pathways, a colocalization analysis was then performed. A pharmacological study was performed to investigate their potential as therapeutic targets for hematological malignancies.

## Methods

### Data sources

We identified cis-single-nucleotide polymorphisms (cis-SNPs) linked to plasma protein levels from two extensive GWAS, the UK Biobank Pharma Proteomics Project (UKB-PPP) 8 and the deCODE Health Study 9. The genetic variation data pertaining to 27 hematological malignancies was acquired through the FinnGen consortium (https://www.finngen.fi/en/access_results). The genome-wide association study on six hematological malignancies was derived from the UK Biobank study (http://www.nealelab.is/uk-biobank/).

### Selection of instrumental variables (IVs)

The plasma protein SNPs that satisfied the GWAS testing P value threshold (< 1×10^-5^) were identified.^10^ All the IVs were categorized according to a linkage disequilibrium (LD) threshold (r^2^ < 0.001) within a distance of 10,000 kilobases (kb), utilizing the 1000 Genomes European reference panel on an individual basis. In instances of palindrome SNPs, the forward allele was established utilizing allele frequency data 11. GWAS of levels of circulating proteins could identify protein quantitative trait loci (pQTLs). Cis-pQTLs were characterized as SNPs located within 1 MB of the gene that encodes the protein, with linkage disequilibrium assessed using the 1000 Genomes European panel 11. To reduce the influence of weak instrumental bias, we calculated the F statistic for each SNP and excluded those with a F statistic lower than 10 12. SNPs with minor allele frequency (MAF)≤0.01 were removed. With the use of the Olink technology, the UKB-PPP was able to perform proteome profiling on blood plasma samples from 54,306 individuals, which resulted in the collection of information for 2,923 proteins 8. During the Mendelian randomization investigation with two samples, we used index cis-SNPs as instrumental variables for a total of 2,112 proteins 8. The deCODE Health project evaluated 4,907 aptamers in a group of 35,559 Icelanders using the SomaScan platform 9. The results of this evaluation were used to produce index cis-SNPs for 1,970 plasma proteins. Each of the two examinations revealed that a total of 999 proteins had index cis-SNPs that overlapped with one another.

### Mendelian randomization (MR)

Multiple MR techniques were used in order to assess the causal link that exists between the exposure and the result. The primary technique of MR analysis that was used was known as the inverse variance weighting (IVW)^13^ approach. When only one SNP instrument was available, the method of causal estimation was the Wald method 14. Following the use of Bonferroni adjustment for multiple testing, the combined relationship was shown to be statistically significant with a *p* value of less than 1.62×10^-5^ (0.05/3083 proteins). However, in order to explore more common plasma proteins of potential hematological malignancies, we set the *P* value at four levels. Level 1: *P* value is less than 1.62×10^-5^, denoted as “* * * *”; Level 2: *P* value ranges from 1.62×10^-5^ to 5×10^-4^, denoted as “* * *”; Level 3: *P* value ranges from 5×10^-4^ to 5×10^-3^, denoted as “* *”; Level 4: *P* value ranges from 5×10^-3^ to 5×10^-2^, denoted as “*”. Sensitivity analysis was employed to estimate pleiotropy and heterogeneity. We assessed heterogeneity using Cochrane's IVW Q statistics 15. We evaluated vertical pleiotropy by examining the intercept obtained from the MR-Egger regression. The MR analyses were carried out using the “TwoSampleMR” software version 0.5.10^16^ packages in R (version 4.3.2).

### Colocalization analysis

We conducted colocalization analysis to see whether linkage disequilibrium accounted for the protein relationships reported with hematological malignancies. The study was conducted using a Bayesian framework, which took into consideration the evidence for five hypotheses that were incompatible with one another 17. For every hypothesis test (H0, H1, H2, H3, and H4), a posterior probability is given to the hypothesis being tested. Under the condition that the posterior probability for shared causal changes (PH4) was more than 0.75, it was determined that two signals had strong evidence for colocalization. It was revealed that the medium colocalization indication lies between 0.5 and 0.75 for PH4. For the purpose of this study, the coloc tool in the R programming language (version 4.4.1) was used.

### Protein-protein interaction (PPI) and functional enrichment analysis

Through the use of the Search Tool for the Retrieval of Interacting Genes (STRING, Version 11.5, https://string-db.org/), PPI networks were built in order to investigate the interactions that occurred between the MR-prioritized proteins. In addition, STRING was used to perform pathway analyses based on the Kyoto Encyclopedia of Genes and Genomes (KEGG) in order to study the possibly enriched pathways that are associated with these proteins.

### Mapping MR-prioritized proteins to drug targets

Plasma proteins are a rich therapeutic target reservoir. The list of druggable genes discovered by Finan et al. 18 was compared to the MR-prioritized proteins to see whether they overlap. Finan et al. 18 discovered 4,479 drugged or druggable genes and divided them into three groups by drug development level. Tier 1 has 1427 genes for effectiveness targets of licensed small compounds, biotherapeutic medicines, and clinical drug candidates. Tier 2 includes 682 genes with demonstrated bioactive drug-like small-molecule binding partners and ≥50% identity (over 75% sequencing) with authorized drug targets. Tier 3 contains 2,370 genes that encode secreted or extracellular proteins, are distantly related to known drug targets, and belong to key druggable gene families missing from tiers 1 or 2. Tier 3A prioritizes genes within ±50 kbp of GWAS SNPs and extracellular locations, whereas Tier 3B includes additional genes. MR-prioritized proteins were annotated as therapeutic targets by development phase using the Therapeutic Target Database (http://db.idrblab.net/ttd/). The database has 3,578 therapeutic targets, including 498 successful, 1,342 clinical trials, 185 preclinical/patented, and 1,553 research targets 19. This analysis focuses on target type, drug connected to target, and illness treated by treatment.

## Results

**Figure 1:**
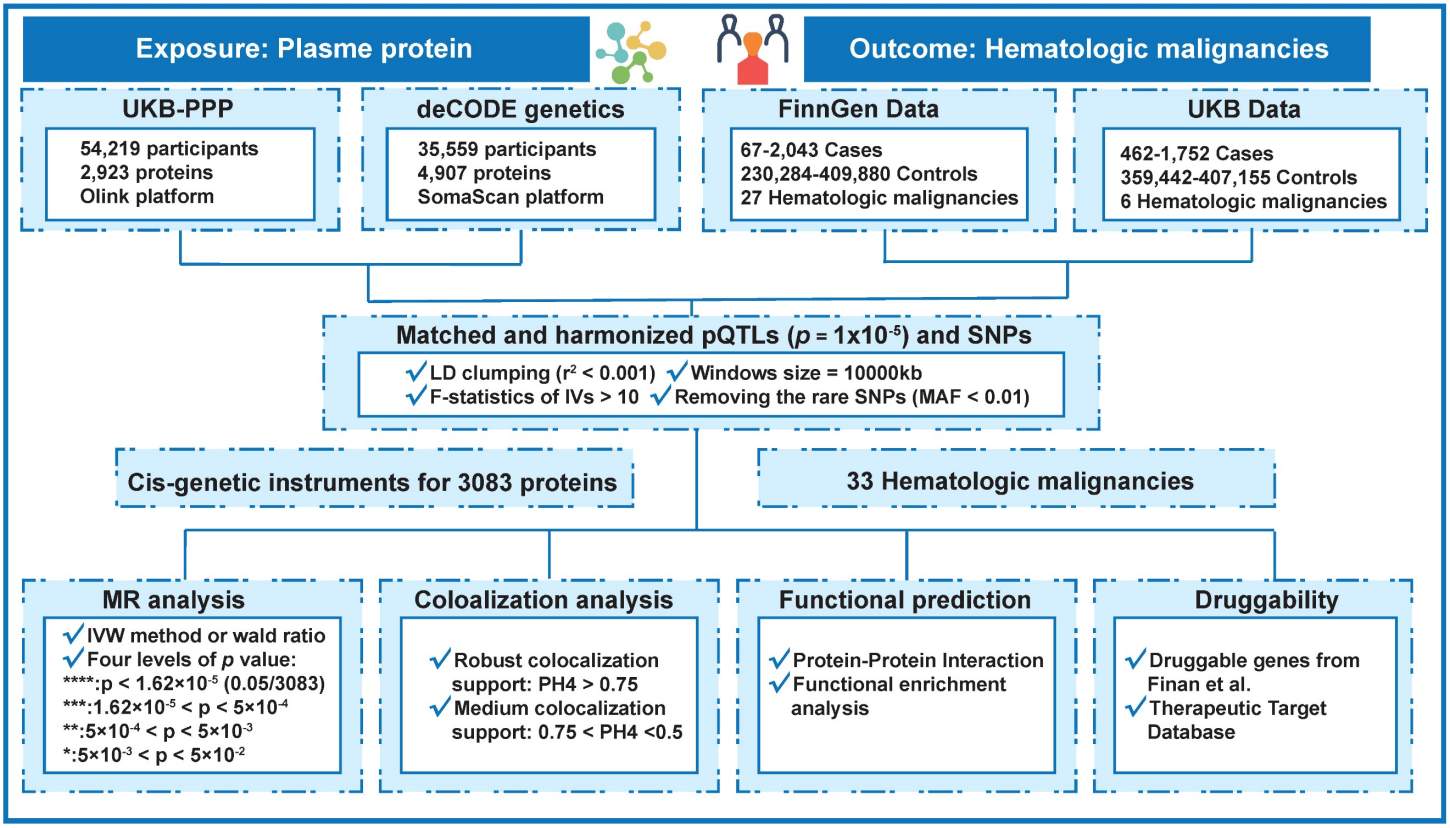
Flowchart of the MR-based analytical framework for evaluating the effect of plasma proteome on 33 hematologic malignancies. UKB-PPP, UK Biobank Pharma Proteomics Project; pQTLs, protein quantitative trait loci; SNP, single-nucleotide polymorphisms; LD, linkage disequilibrium; MAF, minor allele frequency; MR, Mendelian randomization; IVW, inverse variance weighting.

A summary of the conceptual framework of the research is shown in Figure 1. Each and every analysis made use of the data at the summary level that is shown in Table 1. The MR analysis included 1,970 proteins (Table S1) from the deCODE project and 2,112 proteins (Table S2) from the UKB-PPP collaboration; both sets of proteins were analyzed. Every single piece of outcome data showed that the least F statistic for the genetic instruments that were used was more than 10.

### Estimating the effects of plasma proteins on 33 hematological tumors by using MR identified 86 plasma proteins

**Figure 2:**
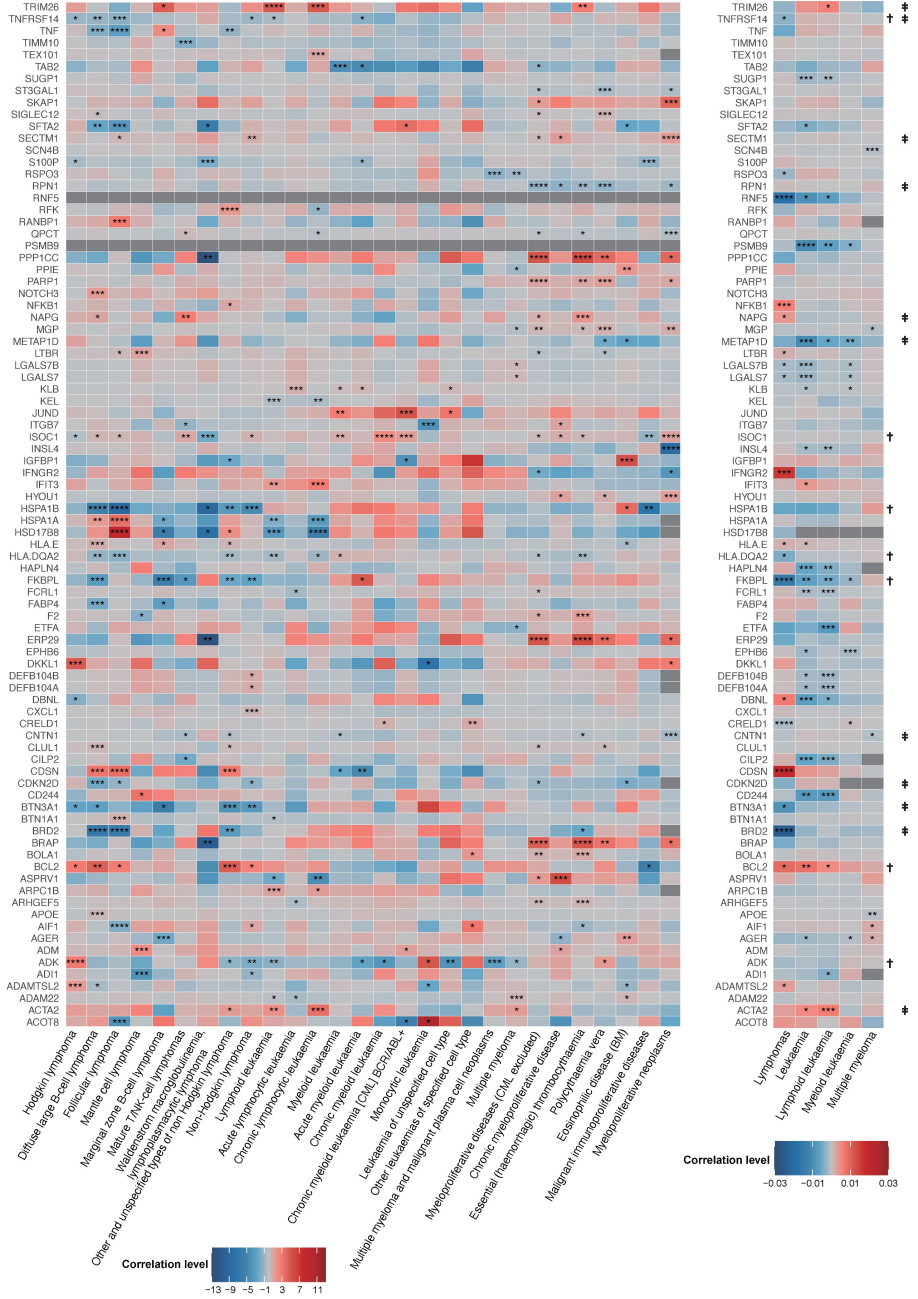
MR analysis on the associations of plasma protein with 33 hematological tumors. The gray squares in indicate missing data. * * * *, p < 1.62×10^-5^; * * *, 1.62×10^-5^ < p < 5×10^-4^; * *, 5×10^-4^ < p < 5×10^-3^; *, 5×10^-3^ < p < 5×10^-2^; †, P value is less than 0.05 in more than or equal to 7 hematological tumors. ‡, In more than or equal to 5 hematological tumors, the P value is less than 0.05, and the β value is greater than 0 or less than 0 at the same time.

The overview of the findings from the examinations of thirty-three distinct hematologic cancers is shown in Figure 2 and Table S3. When the *p*-value of a protein is less than 5×10^-4^, we consider it to be a candidate plasma protein. This methodology allows us to investigate a greater number of prospective plasma proteins that are often associated with hematological malignancies. The genetically predicted amounts of 86 proteins were shown to have a substantial association with the probability of hematological tumors, as shown in Figure 2 of the combined analysis of two outcomes data. We found that some proteins have causal associations in multiple hematological tumors. Our findings indicate that certain plasma proteins exhibit causative roles in various hematological tumors, specifically ISOC1 in 14 tumors, ADK in 11 tumors, FKBPL in 10 tumors, and BCL2 in 9 tumors. In addition, we found that some plasma proteins play the same role in many blood tumors. ACTA2 serves as a risk factor across all seven types of blood tumors. CNTN1 serves as a protective factor in six types of hematological tumors (Figure 2 and Table S3).

### 27 plasma proteins were verified by colocalization evidence

**Figure 3:**
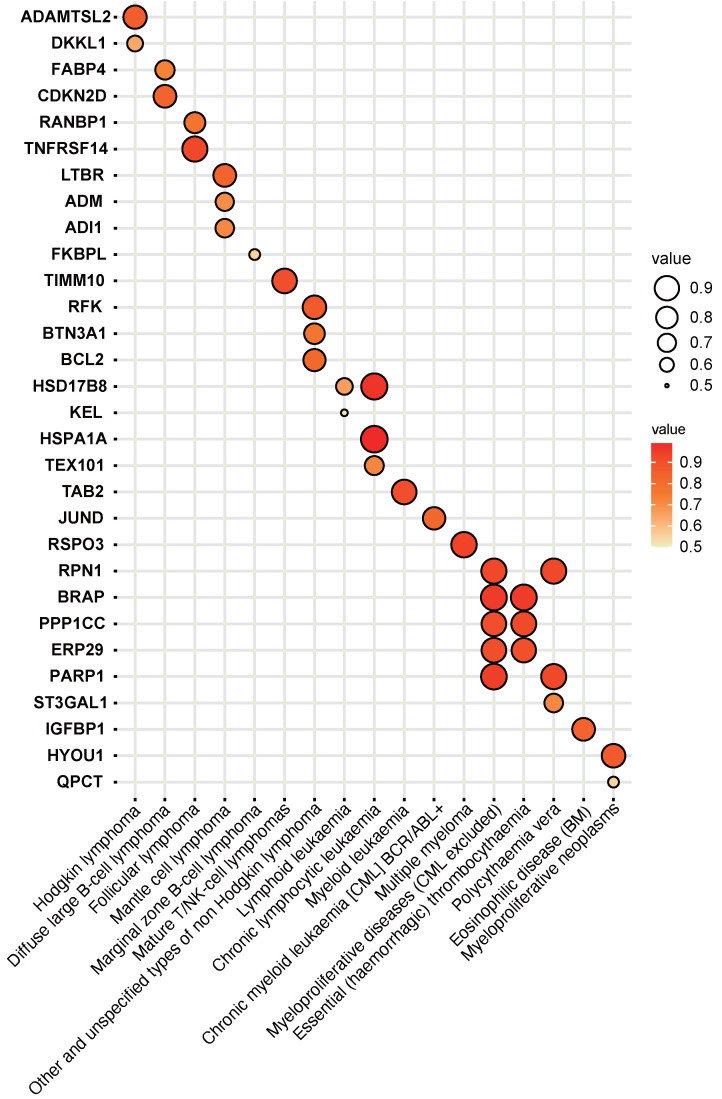
Colocalization analysis between plasma protein (P value of MR < 5×10^-4^) and hematologic malignancies.

27 plasma proteins were verified by colocalization evidence. 18 proteins demonstrated strong support of colocalization analysis (PH4 > 0.75) out of 86 MR-identified proteins in connection to hematological tumors (P value of MR < 5×10^-4^) (Figure 3 and Table S4). Nine proteins obtained medium support of colocalization analysis (0.75 < PH4 < 0.5) (Figure 3 and Table S4). Some diseases have colocalization with multiple other diseases. For example, myeloproliferative diseases (excluding chronic myeloid leukemia, CML) have the most colocalized genes, including RPN1, BRAP, PPP1CC, ERP29, and PARP1. Moreover, myeloproliferative diseases (excluding CML) and essential (haemorrhagic) thrombocythaemia have three identical colocalized genes, namely BRAP, PPP1CC, and ERP29. In addition, certain genes have colocalization with multiple diseases. For instance, HSD17B8 has colocalization with lymphoid leukemia and chronic lymphocytic leukemia; RPN1 has colocalization with myeloproliferative diseases (excluding CML) and polycythaemia vera; and PARP1 has colocalization with myeloproliferative diseases (excluding CML) and polycythaemia vera.

### PPI networks and KEGG pathway of the 86 MR-prioritized proteins

**Figure 4:**
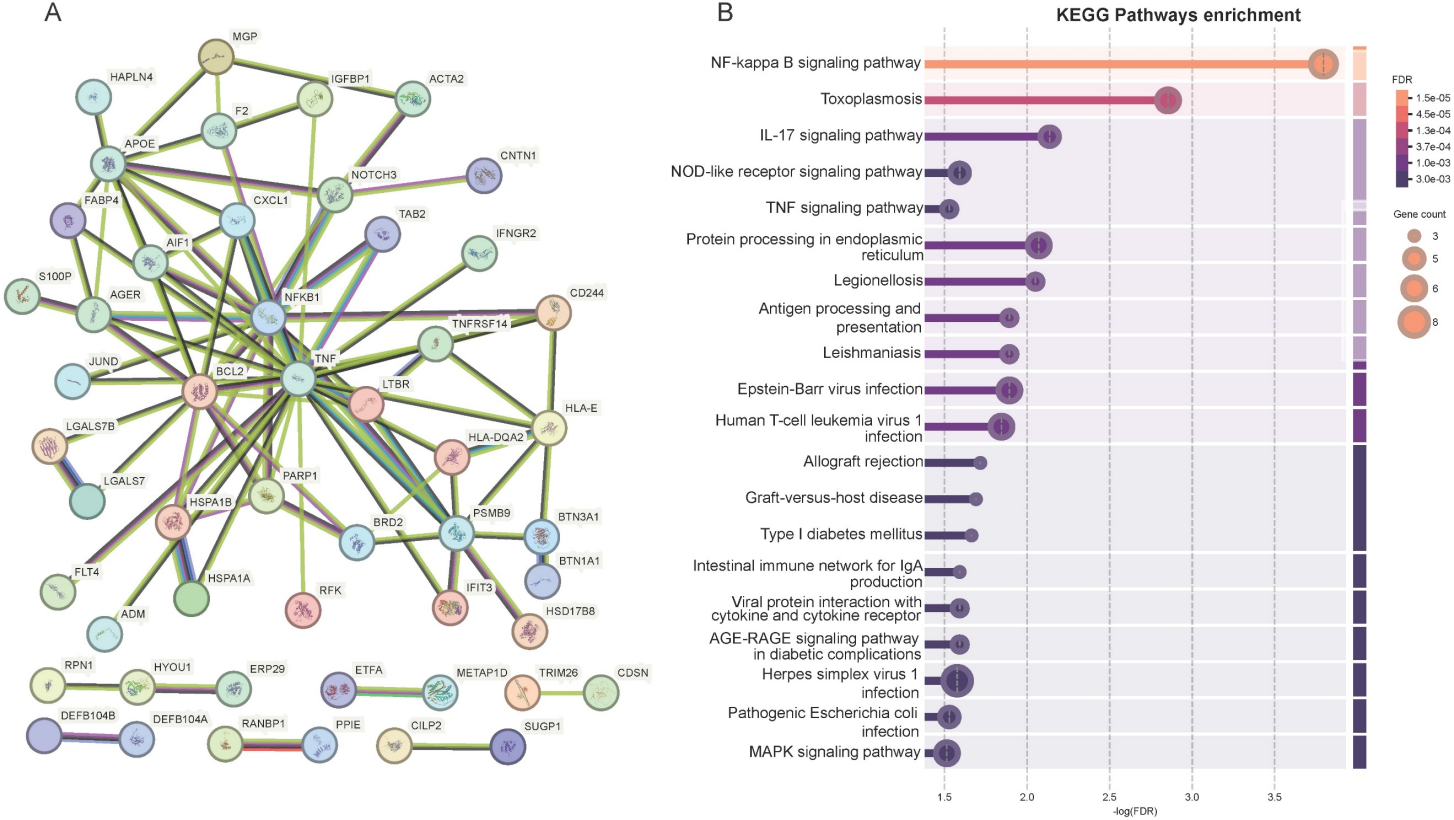
Protein-protein interaction networks (A) and KEGG Pathways enrichment (B) of the 86 MR-prioritized proteins.

PPI and pathway studies were carried out in order to get a better knowledge of the etiology of hematological tumors and to gain a better understanding of the association between 86 MR-prioritized proteins and their enriched activities (Figure 4A, Table S5). The PPI network comprised 87 nodes and produced 92 edges, significantly exceeding the anticipated 58 edges based on an interaction score threshold of 0.4 (medium confidence) (enrichment p-value: 8.86×10^-5^) (Figure 4A, Table S5). By KEGG enrichment analysis, 86 proteins were mainly enriched in the NF-kappa B signaling pathway, the IL-17 signaling pathway, the NOD-like receptor signaling pathway, the TNF signaling pathway, and so on (Figure 4B, Table S6).

### Evaluating the drug targets of the 86 MR-prioritized proteins

The assessment of human proteins utilizing MR evidence concentrated on their viability as therapeutic targets and their potential for drug development. We first compared the MR-prioritized proteins with the druggable genes identified by Finan et al 18. Among the 86 proteins analyzed, 51 exhibited druggable targets, categorized as follows: 9 in tier 1, 8 in tier 2, 18 in tier 3A, and 16 in tier 3B (Table 2). Utilizing the Therapeutic Target Database, a total of 26 proteins were identified as targets for existing or potential drugs. Among these, 6 were classified as successful targets, 13 as clinical trial targets, 6 as literature-reported targets, and 1 as a discontinued target (Table 2; Table S7).

## Discussion

This MR study investigated the relationships between 3,083 plasma proteins and the risk of 33 hematological tumors, complemented by a colocalization analysis. We identified 86 plasma proteins potentially causally associated with 33 hematological tumors, of which 27 proteins demonstrated colocalization support. Function prediction associated with these proteins enriched several pathways, including the NF-kappa B signaling pathway, IL-17 signaling pathway, NOD-like receptor signaling pathway, and TNF signaling pathway. The therapeutic potential of 86 proteins and their pharmaceutical properties were assessed.

There are a few proteins that have been found to be related to hematologic cancers, and these proteins were prioritized by our MR analysis in our research that used cis-pQTL. For example, BCL2, a key regulator of apoptosis, is frequently overexpressed in hematological tumors, contributing to chemotherapy resistance and poor prognosis 20. Targeting BCL2 has emerged as a promising therapeutic approach, with BH3 mimetics showing particular potential 21, 22.

Venetoclax, the first FDA-approved BCL2 inhibitor, has demonstrated significant efficacy in chronic lymphocytic leukemia and other hematologic cancers 23. However, response to BCL2 inhibition varies across different malignancies, highlighting the need for biomarkers to predict treatment outcomes 23. Ongoing research focuses on developing new BCL2 family inhibitors, optimizing combination therapies, and understanding resistance mechanisms 24. As our understanding of BCL2 biology and apoptosis signaling improves, targeted therapies are expected to play an increasingly important role in treating hematological tumors 22, 25. NF-κB, a crucial transcription factor family including NFKB1, plays a significant role in hematological tumors through its regulation of cell proliferation, apoptosis, and inflammation 26. Constitutive NF-κB activation is common in these malignancies, contributing to enhanced cell survival and proliferation 27. This activation can result from genetic alterations, microenvironmental factors, or chronic signaling 27. NF-κB's pro-survival properties rely on the expression of anti-apoptotic molecules 28. Targeting NF-κB and its regulators has emerged as a promising therapeutic approach for hematological tumors 29, 30. PARP1 overexpression has been observed in various hematological tumors, suggesting its potential as a therapeutic target 31. PARP inhibitors (PARPi) have shown promise in treating acute myeloid leukemia (AML) and other blood cancers, particularly in cases with specific genetic alterations such as RUNX1-RUNX1T1, PML-RARA, FLT3, and IDH1/2 mutations 32. MYC-driven multiple myeloma cells exhibit increased sensitivity to PARPi 33. Combining PARPi with other therapies, such as HDAC inhibitors or chemotherapeutic agents, has demonstrated synergistic effects 34. TNFRSF14 mutations and 1p36 deletions are frequent genetic alterations in follicular lymphoma (FL), occurring in 18-40% of cases 35, 36. These aberrations are associated with worse prognosis, increased risk of transformation, and inferior overall and disease-specific survival, particularly when both mutations and deletions are present 35, 37. TNFRSF14 alterations reduce HVEM expression, enhancing the alloantigen-presenting capacity of lymphoma B cells and increasing the risk of acute graft-versus-host disease in allogeneic stem cell transplantation 38. High TNFRSF14 expression correlates with poor outcomes and activation of the NF-kB pathway 37. In contrast, high expression of BTLA, which interacts with TNFRSF14, is associated with better overall survival 37. These findings highlight the importance of the BTLA-TNFRSF14 immune modulation pathway in FL pathobiology and prognosis. BRD2, a member of the bromodomain and extra-terminal (BET) family, plays a crucial role in hematological tumors. It promotes B-cell expansion and mitogenesis by regulating cyclin A expression 39. Overexpression of BRD2 in transgenic mice leads to B-cell lymphoma and leukemia development 40. BRD2 is also a critical mediator for STAT5 activity in leukemias and lymphomas 41. BET inhibitors, such as JQ1, show promise in treating hematological tumors by targeting BRD proteins, including BRD2 and BRD4 42. In acute myeloid leukemia (AML), FABP4 promotes aggressiveness through a vicious loop with DNA methyltransferase 1 (DNMT1), enhancing aberrant DNA methylation 43, 44. FABP4 inhibition suppresses AML progression and induces leukemia regression in mouse models 45. Bone marrow adipocytes support AML proliferation through FABP4-mediated fatty acid transfer 45. FCRL1, a member of the Fc receptor-like family, is predominantly expressed on B cells and plays a role in B cell regulation and malignancies 46. It shows high expression in various B-cell non-Hodgkin lymphomas, chronic lymphocytic leukemia (CLL), and hairy cell leukemia 47. FCRL1 has been identified as a potential target for immunotoxin therapy in these malignancies 48. Its expression correlates with IGHV mutation status in CLL, making it a valuable prognostic marker 49. FCRL1 promotes B cell proliferation and survival through the PI3K/AKT and NF-κB pathways 50. Recent studies have highlighted the importance of IFIT family proteins in hematological tumors. IFIT1, IFIT2, IFIT3, and IFIT5 are overexpressed in acute myeloid leukemia (AML) patients, with higher levels of IFIT2, IFIT3, and IFIT5 predicting poor prognosis 51. IFIT1 and IFIT3 have been implicated in proptosis induction in myeloma and leukemia cells 52.

In addition to some proteins that have been identified with hematological tumors, our research also found some plasma proteins that are related to most hematological tumors. For example, ISOC1 is causally associated with 14 hematological tumors. While ISOC1 promotes cell proliferation and tumor growth in pancreatic cancer 53 and lung cancer 54, it acts as a tumor suppressor in hepatocellular carcinoma 55. ADK is causally associated with 14 hematological tumors. Dysregulation of ADK isoforms, particularly overexpression of the nuclear long isoform (ADK-L), has been observed in breast cancers, contributing to tumor growth and metastasis 56. ADK deficiency increases susceptibility to carcinogens in the liver 57. ADK's involvement in DNA methylation suggests an epigenetic role in cancer pathology 58. FKBPL is causally associated with 11 hematological tumors. FKBPL, a novel member of the immunophilin family, has emerged as a potential cancer biomarker and therapeutic target. It plays crucial roles in steroid receptor signaling, particularly for estrogen, androgen, and glucocorticoid receptors 59. High levels of FKBPL are associated with increased survival and improved response to endocrine therapy in breast cancer patients 60.

Our study highlights key targets like BCL2, NFKB1, and TNFRSF14, which regulate apoptosis, inflammation, and immune responses. While promising, their systemic inhibition poses toxicity risks. For example, BCL2 inhibitors (e.g., venetoclax) show efficacy in hematologic malignancies but cause severe neutropenia due to broad lymphoid dependency 61. Similarly, NF-κB suppression may impair antimicrobial immunity 62, and PARP inhibitors induce hematologic toxicity from ubiquitous DNA repair roles 63. Resistance remains a major hurdle. BCL2-targeted therapies fail via mutations (e.g., G101V) or compensatory MCL1 upregulation 64, while PARP inhibitor resistance arises through BRCA/PALB2 mutation reversal 65. Combinatorial approaches (e.g., PARP + ATR inhibitors) could overcome escape mechanisms 66. TNFRSF14 exemplifies context-dependency: loss drives lymphoma progression yet may enhance immunogenicity in specific subtypes, necessitating biomarker-driven stratification 38. Emerging strategies (PROTACs, nanobodies) may bypass these limitations. Future work should: 1) Validate lead targets (PARP1, TNFRSF14) using PDX models and CRISPR screens; 2) Develop companion diagnostics via multi-omic profiling; 3) Optimize delivery systems (nanoparticles) to enhance specificity. Drug repurposing (e.g., NF-κB inhibitors) offers accelerated translation potential.

In conclusion, following the MR findings, we performed a series of subsequent analyses. Our study indicates that fewer than 50% of the associations demonstrate robust evidence of colocalization that supports causality. The assumption in Bayesian colocalization approaches that only one association signal exists per region may not accurately reflect reality, potentially leading to an underestimation of colocalization. Protein-protein interaction and pathway enrichment analyses were performed to investigate the relationships and functions of the identified proteins. Evaluation of drug targets was conducted for proteins with MR evidence, aiming to priorities drug discovery and facilitate the repurposing of existing drugs for hematological tumors.

## Supplementary Material

Supplementary tables.

## Figures and Tables

**Table 1:** Characteristics of exposures and outcome.

Variable	Source	Cases	Controls
**Exposure**			
4,907 plasma protein (pQTLs)	deCODE Health study	35,559	/
2,923 plasma protein (pQTLs)	UK Biobank Pharma Proteomics Project (UKB-PPP)	54,219	/
**Outcome**			
Hodgkin lymphoma	FinnGen data CD2_HODGKIN_LYMPHOMA_EXALLC	846	324,650
Diffuse large B-cell lymphoma	FinnGen data C3_DLBCL_EXALLC	1,050	314,193
Follicular lymphoma	FinnGen data CD2_FOLLICULAR_LYMPHOMA_EXALLC	1,181	324,650
Mantle cell lymphoma	FinnGen data C3_MANTLE_CELL_LYMPHOMA_EXALLC	210	314,193
Marginal zone B-cell lymphoma	FinnGen data C3_MARGINAL_ZONE_LYMPHOMA_EXALLC	202	314,193
Mature T/NK-cell lymphomas	FinnGen data CD2_TNK_LYMPHOMA_EXALLC	363	324,650
Waldenstrom macroglobulinemia, lymphoplasmacytic lymphoma	FinnGen data C3_MACROGLOBULINEMIA_EXALLC	88	314,193
Other and unspecified types of non Hodgkin lymphoma	FinnGen data CD2_NONHODGKIN_NAS_EXALLC	1,171	324,650
Non-Hodgkin lymphoma	FinnGen data C3_NONHODGKIN_EXALLC	1,072	314,193
Lymphoid leukaemia	FinnGen data CD2_LYMPHOID_LEUKAEMIA_EXALLC	1,617	324,650
Acute lymphocytic leukaemia	FinnGen data C3_ALL_EXALLC	197	314,192
Chronic lymphocytic leukaemia	FinnGen data C3_CLL_EXALLC	668	314,189
Myeloid leukaemia	FinnGen data CD2_MYELOID_LEUKAEMIA_EXALLC	734	324,650
Acute myeloid leukaemia	FinnGen data C3_AML_EXALLC	244	314,192
Chronic myeloid leukaemia	FinnGen data C3_CML_EXALLC	115	314,192
Chronic myeloid leukaemia [CML] BCR/ABL+	FinnGen data CML	258	409,880
Monocytic leukaemia	FinnGen data CD2_MONOCYTIC_LEUKAEMIA_EXALLC	85	324,650
Leukaemia of unspecified cell type	FinnGen data CD2_LEUKAEMIA_NAS_EXALLC	239	324,650
Other leukaemias of specified cell type	FinnGen data CD2_OTHER_LEUKAEMIA_SPECIFIED_EXALLC	67	324,650
Multiple myeloma and malignant plasma cell neoplasms	FinnGen data CD2_MULTIPLE_MYELOMA_PLASMA_CELL_EXALLC	1,337	324,650
Multiple myeloma	FinnGen data C3_MULT_MYELOMA_EXALLC	623	314,185
Myeloproliferative diseases (CML excluded)	FinnGen data MYELOPROF_NONCML	2,043	409,880
Chronic myeloproliferative disease	FinnGen data CHRONMYELOPRO	354	409,880
Essential (haemorrhagic) thrombocythaemia	FinnGen data THROMBOCYTAEMIA	1,062	313,473
Polycythaemia vera	FinnGen data POLYCYTVERA	1,004	313,577
Eosinophilic disease (BM)	FinnGen data ESOSINOPHIL_DISEASE	441	230,284
Malignant immunoproliferative diseases	FinnGen data CD2_IMMUNOPROLIFERATIVE_EXALLC	250	324,650
Lymphomas	UK Biobank data	1,752	359,442
Leukaemia	UK Biobank data	1,260	372,016
Lymphoid leukaemia	UK Biobank data	760	372,016
Myeloid leukaemia	UK Biobank data	462	372,016
Multiple myeloma	UK Biobank data	601	372,016
Myeloproliferative neoplasms	UK Biobank data	1,086	407,155

**Table 2:** List of the 86 MR-prioritized proteins that were drug targets or to be druggable.

Protein	Druggability tier	Target type	Protein	Druggability tier	Target type
ADK	Tier 2	Clinical trial Target	HYOU1	Tier 3B	/
ADM	Tier 3A	Clinical trial Target	IFNGR2	Tier 1	Successful Target
AGER	Tier 3A	Clinical trial Target	IGFBP1	Tier 2	Discontinued Target
AIF1	/	Literature-reported target	INSL4	Tier 3A	/
APOE	Tier 3A	Clinical trial Target	ITGB7	Tier 1	Successful Target
BCL2	Tier 1	Successful Target	KEL	Tier 3B	/
BOLA1	Tier 3A	/	KLB	/	Clinical trial Target
BRD2	Tier 2	Clinical trial Target	LGALS7	Tier 3B	/
BTN1A1	Tier 3A	/	LGALS7B	Tier 3B	/
BTN3A1	Tier 3B	/	LTBR	Tier 1	Clinical trial Target
CD244	Tier 3A	/	MGP	Tier 3B	/
CILP2	Tier 3A	/	NFKB1	Tier 1	Successful Target
CLUL1	Tier 3B	Literature-reported target	NOTCH3	Tier 3B	Clinical trial Target
CNTN1	Tier 3A	/	PARP1	Tier 1	Successful Target
CXCL1	Tier 3B	Literature-reported target	PPIE	Tier 2	/
DEFB104A	Tier 3B	/	PSMB9	Tier 3B	Literature-reported target
DEFB104B	Tier 3B	/	QPCT	Tier 2	Clinical trial Target
DKKL1	Tier 3A	/	RPN1	Tier 3A	/
EPHB6	Tier 1	Literature-reported target	RSPO3	Tier 3A	Clinical trial Target
ERP29	Tier 3A	/	SECTM1	Tier 3B	/
F2	Tier 1	Successful Target	SFTA2	Tier 3A	/
FABP4	Tier 2	/	SIGLEC12	Tier 3B	/
FCRL1	Tier 3A	/	ST3GAL1	Tier 3B	/
HAPLN4	Tier 3A	/	TEX101	Tier 3B	/
HLA-DQA2	Tier 3A	/	TNF	Tier 1	Clinical trial Target
HSPA1A	Tier 2	Clinical trial Target	TNFRSF14	Tier 3A	Literature-reported target
HSPA1B	Tier 2	Clinical trial Target			

## Abbreviations

GWASs: Genome-wide association studies
UKB-PPP: UK Biobank Pharma Proteomics Project
MR: Mendelian randomization
IVs: instrumental variables
SNPs: single-nucleotide polymorphisms
LD: linkage disequilibrium
IVW: inverse variance weighting
KEGG: Kyoto Encyclopedia of Genes and Genomes
OR: Odds Ratio
PPI: Protein-protein interaction
